# New Poisson–Sch type inequalities and their applications in quantum calculus

**DOI:** 10.1186/s13660-018-1735-6

**Published:** 2018-07-03

**Authors:** Tao Liu, Xinjuan Chen, Yifan Xing

**Affiliations:** 1 School of Mathematics and Computer Science, Panzhihua University, Panzhihua, China; 2 School of Mechanotronics and Vehicle Engineering, Qingyuan Polytechnic, Qingyuan, China; 3 Shenzhen Quantum Wisdom Culture Development Company, Shenzhen, China; 4 Centre for Quantum Technologies, National University of Singapore, Singapore, Singapore

**Keywords:** Poisson–Sch type inequality, Schrödinger operator, Modified Poisson–Sch kernel function

## Abstract

The Poisson type inequalities, which were improved by Shu, Chen, and Vargas-De-Teón (J. Inequal. Appl. 2017:114, 2017), are generalized by using Poisson identities involving modified Poisson kernel functions with respect to a cone. New generalizations of improved Poisson–Sch type inequalities are obtained by using the generalized Montgomery identity associated with the Schrödinger operator. As applications in quantum calculus, we estimate the size of weighted Schrödingerean harmonic Bergman functions in the upper half space.

## Introduction

The Poisson–Sch inequality problem has many applications, e.g., second-order irreversible reactions, obstacle problems, the diffusion problem involving Michaelis–Menten, and reservoir simulation, see, for example, [11, 16–18] and the references therein for details. In recent years, various extensions and generalizations of the classical variational inequality models and complementarity problems have emerged in mechanics, nonlinear programming, physics, optimization and control, economics, transportation, finance, structural, elasticity, and applied sciences; see [7, 12, 17, 18] and the references therein for more details. And hence there are a number of numerical methods, such as descent and decomposition, neutral differential equations, for the solution of Poisson–Sch inequality models and complementarity problems [16, 17].

In general, when this method, or its many Poisson–Sch forms, is used to solve the Poisson–Sch inequality problem, a key element for implementing this is to find the projection operator. And then, based on the assumption of the convex set, the sequence generated by the proposed method converges to the unique solution of the Poisson–Sch inequality problem. However, for some classes of variational inequalities, such as the generalized nonlinear Poisson–Sch inequality systems, there is not a general convergence theorem, owing to the fact that the convex set cannot be built and the projection method is inapplicable [1, 5, 13, 24]. To fix this issue, the auxiliary principle method has been used to the Poisson–Sch inequality problem, the origin of which can be traced back to the reference by Lions and Stampacchia [16]. Moreover, the authors in [12, 20, 22] used an auxiliary principle method to study the existence of a solution of mixed variational inequalities. In recent years, under the frame of the auxiliary principle, some authors, such as Huang [14], Qiao [20], Shu et al. [21], Wang et al. [22], Zhao and Zhang [23], and so on, introduced some interesting iterative algorithms to solve some classes of Poisson–Sch inequality problems, and built the corresponding convergence theorems.

Due to the rapid advancement of computing resource, there is a growing interest in developing parallel algorithms for the simulation of the Poisson–Schinequality problem. However, most approaches for Poisson–Sch inequality problems are based on the sequential iterative method. Motivated and inspired by the references [21, 23], in this paper we introduce and investigate some new Poisson–Sch type inequalities and obtain some applications.

Let $\mathbf{R}_{+}$ be a set of all positive real numbers and $\mathbf{R}^{n-1}$ be the *n*-dimensional Euclidean space, where $n \ge 2$. A point *z* in **H** is denoted by $(z^{\prime }, z _{n})$, where $\mathbf{H} = \mathbf{R}^{n-1}\times \mathbf{R}_{+}$, $z^{\prime }\in \mathbf{R}^{n-1} $, and $z_{n} > 0$.

Let $\zeta >0$ and *h* be a Schwarz function. Then the positive powers of the Laplace operator Δ can be defined by (see, e.g., [10, p. 102])

1 $$ (-\Delta )^{\frac{\tau }{2}}h(z)=\mathfrak{F}^{-1} \bigl( \vert \xi \vert ^{\tau } \hat{h}(\xi ) \bigr) $$

and

$$ \mathfrak{F}h(\xi )=\hat{h}(\xi )= \int_{\mathbf{R}^{n}}h(x)e^{-ix\xi }\,dx. $$

It is well known that the definition (1) can be extended to certain negative powers of −Δ, and we can define

$$ L_{\tau }h=(-\Delta )^{-\frac{\tau }{2}}h=\mathfrak{F}^{-1} \bigl( \vert \xi \vert ^{- \tau }\hat{h} \bigr)\quad (0< \tau < n). $$

If we define the inverse Fourier transform of $\vert \xi \vert ^{-\tau }$ by $L_{\tau }$, then it follows that [9, p. 61]

$$ L_{\tau }(z)=\frac{\gamma_{\tau }}{\vert z \vert ^{n-\tau }}, $$

where $\gamma_{\tau }$ is a certain constant.

Let $0<\tau <n$ and $g=(-\Delta )^{\frac{\tau }{2}}h$. Then it is well known that any Schwartz function *h* can be written as follows:

$$ h(z)=L_{\tau }g(z)=(L_{\tau }\ast g) (z)=\gamma_{\tau } \int_{\mathbf{R} ^{n}}\frac{g(w)}{\vert z-w \vert ^{n-\tau }}\,dw. $$

A time scale is defined by $\mathbb{T}$. Then the operators $\sigma :\mathbb{T}\to \mathbb{T}$ and $\rho :\mathbb{T}\to \mathbb{T}$ are defined as follows:

$$ \sigma (t) =\inf \{ s\in \mathbb{T}:s>t\} $$

and

$$ \rho ( t) =\sup \{ s\in \mathbb{T}:s< t\}, $$

respectively, where $t\in \mathbb{T}$ (see [4, 6, 24]).

Let *a* and *b* be fixed two points in $\mathbb{T}$ satisfying $a\leq b$. The modified Schrödinger equation is defined by

2 $$ l( y) :=- \bigl[ p( t) y^{\Delta }( t) \bigr] ^{\nabla }+q( t) y( t) , \quad t \in [ a,b], $$

where $q:\mathbb{T}\to \mathbb{C}$ is a continuous function, $p:\mathbb{T}\to \mathbb{C}$ is ∇-differentiable on $\mathbb{T}^{k}$, $p(t) \neq 0$ for all $t\in \mathbb{T}$, and $p^{\nabla }:\mathbb{T}_{k} \to \mathbb{C}$ is continuous. The Wronskian of *y*, *z* is defined as

$$ W( y,z) ( t) :=p( t) \bigl[ y(t) z^{\Delta }( t) -y^{\Delta }( t) z(t) \bigr] , $$

where $t\in \mathbb{T}^{\ast }$ (see [8]).

Consider the boundary-value problem defined by

3 $$ l(y)=\lambda y,\quad y\in D, $$

subject to the boundary conditions

4 $$\begin{aligned}& y( b) -hp( b) y^{\Delta }( b) =0,\qquad \operatorname{Im}h>0 , \end{aligned}$$

5 $$\begin{aligned}& \upsilon_{1}y( a) -\upsilon_{2}p( a) y^{\Delta }( a) =\lambda \bigl( \upsilon_{1}'y( a) -\upsilon_{2}'p( a) y^{\Delta }( a) \bigr), \end{aligned}$$

where *λ* is a spectral parameter and $\upsilon_{1},\upsilon _{2},\upsilon_{1}',\upsilon_{2}'\in \mathbb{R}$, and *υ* is defined by

$$ \upsilon := \begin{vmatrix} \upsilon_{1}' & \upsilon_{1} \\ \upsilon_{2}' & \upsilon_{2} \end{vmatrix} = \upsilon_{1}'\upsilon_{2}-\upsilon_{1} \upsilon_{2}'>0. $$

Motivated by this Riesz kernel $L_{\tau }$, we shall introduce the modified Riesz kernel function in **H**. To do this, we first set (see [2])

$$ E_{\tau }(z)= \textstyle\begin{cases} -\log \vert z \vert & \mbox{if } \tau =n=2, \\ \vert z \vert ^{\tau -n} & \mbox{if } 0< \tau < n. \end{cases} $$

We define the modified Riesz kernel $G_{\tau }(z,w)$ by

$$ E_{\tau }(z-w)-E_{\tau } \bigl(z-w^{\ast } \bigr), $$

where $z\neq w$, $0<\tau \leq n$ and ^∗^ denotes reflection in the boundary plane *∂***H** just as $w^{\ast }=(w_{1},w _{2},\ldots ,w_{n-1},-w_{n})$.

Let $\zeta > 0$, $0< p < \infty $, $\Omega \subset \mathbf{R}^{n}$, and $1/p+1/q=1$. Then the weighted harmonic space $\aleph^{p} _{\zeta }( \Omega ) $ can be defined by

$$ \Vert u \Vert _{\aleph^{p}_{\zeta }(\Omega )}:= \biggl( \int_{ \Omega } \bigl\vert u(z) \bigr\vert ^{p}\,d \wp_{\zeta }(z) \biggr) ^{\frac{1}{q}}< \infty , $$

where *u* are real-valued harmonic functions on Ω, $d\wp_{ \zeta }(z)=\operatorname{dist}(z,\partial \Omega )^{\zeta }\,dz$. Let $\operatorname{dist}(z,\partial \Omega )$ be the distance from *z* to *∂*Ω and *dz* denote the Lebesgue measure on $\mathbf{R}^{n}$ (see [15, 19]). Put $\aleph^{p}_{\zeta }=\aleph^{p}_{\zeta }(\mathbf{H})$. Then we can check that $dV_{\zeta }(z) = z^{\zeta }_{n}\,dz$ on **H**.

## Preliminary results

In this section, we further present some basic definitions, concepts, and some fundamental results that will be used later.

### Definition 2.1

*A* mapping $\mathfrak{T}:\mathbf{H}\rightarrow \mathbf{H}$ goes by the name of (see [3]):

(i) Nonexpansive, if
  $$\begin{aligned} \Vert \mathfrak{T}z-\mathfrak{T}w\Vert \le \Vert z-w\Vert \end{aligned}$$
  for all $z,w\in \mathbf{H}$.
(ii) Firmly nonexpansive, if
  $$\begin{aligned} \Vert \mathfrak{T}z-\mathfrak{T}w\Vert \le \langle z-w, \mathfrak{T}z- \mathfrak{T}w \rangle \end{aligned}$$
  for all $z,w \in \mathbf{H}$.
(iii) Contractive on *x*, if there exists $0<\zeta <1$ such that
  $$\begin{aligned} \Vert \mathfrak{T}z-\mathfrak{T}w\Vert \le \zeta \Vert z-w\Vert \end{aligned}$$
  for all $z,w\in \mathbf{H}$.
(iv) Monotone, if
  $$\begin{aligned} \langle \mathfrak{T}z-\mathfrak{T}w,z-w \rangle \ge 0 \end{aligned}$$
  for all $z,w \in \mathbf{H}$.
(v) *κ*-inverse strongly monotone, if there exists $\kappa >0$ such that
  $$\begin{aligned} \kappa \Vert \mathfrak{T}z-\mathfrak{T}w\Vert ^{2}\leq \langle \mathfrak{T}z- \mathfrak{T}w,z-w \rangle \end{aligned}$$
  for all $z,w\in \mathbf{H}$.

Define

6 $$\begin{aligned} P_{z}(w):=P(z,w)=\frac{z_{n}+w_{n}}{n\vert z-\overline{w} \vert ^{n}}, \end{aligned}$$

where $w \in \overline{\mathbf{H}}$ and $\overline{w}=(w^{\prime }, -w _{n})$. We call it the general Poisson kernel.

It follows from (6) that

7 $$\begin{aligned} D_{z}^{\vec{\kappa }}P(z,w):=D_{z_{1}}^{\kappa_{1}} \cdots D_{z_{1}} ^{\kappa_{1}}P(z,w) =\frac{f(z-\overline{w})}{\vert z-\overline{w} \vert ^{n+2\vert \vec{\kappa } \vert +1}} \end{aligned}$$

for

$$ \vec{\kappa }=\kappa_{1}+\kappa_{2}+\cdots + \kappa_{n} $$

and

8 $$\begin{aligned} \int_{\partial \mathbf{H}}P(z,w)\,dw^{\prime }=1 \end{aligned}$$

for each $z\in \mathbf{H}$ and for every $w \in \overline{\mathbf{H}}$, where *f* is a homogeneous polynomial of degree $\vert \vec{\kappa } \vert +2$ (see [2] for more details).

The following lemmas are called Green–Sch type estimates of Green–Sch functions $G_{\tau }(\cdot ,\cdot )$ (see [21, 22]).

### Lemma 2.1

*Let* $0<\zeta \leq n$. *Then*

$$ G_{\tau }(z,w)\approx M\frac{z_{n}w_{n}}{\vert z-w \vert ^{n-\zeta +2}}. $$

### Lemma 2.2

1. $$ \bigl\vert \mathcal{C}_{k}^{\omega }(t) \bigr\vert \leq \mathcal{C}_{k}^{\omega }(1)=\frac{ \Gamma (2\omega +k)}{\Gamma (2\omega )\Gamma (k+1)}, $$
  *where* $\vert t \vert \leq 1$;
2. $$ \frac{d}{dt}\mathcal{C}_{k}^{\omega }(t)=2\omega \mathcal{C}_{k-1} ^{\omega +1}(t), $$
  *where* $k \geq 1$;
3. $$ \sum_{k=0}^{\infty } \mathcal{C}_{k}^{\omega }(1)r^{k}=(1-r)^{-2 \omega }; $$
4. $$ \bigl\vert \mathcal{C}^{\frac{n-\zeta }{2}}_{k} (t)-\mathcal{C}^{\frac{n-\zeta}{2}}_{k} \bigl(t^{\ast } \bigr) \bigr\vert \leq (n-\zeta )\mathcal{C}^{ \frac{n-\zeta +2}{2}}_{k-1} (1) \bigl\vert t-t^{\ast } \bigr\vert , $$
  *where* $\vert t \vert \leq 1$ *and* $\vert t^{\ast } \vert \leq 1$.

## Main results and their applications

In this section, we present the proposed parallel iterative method with auxiliary principle for the generalized Schrödinger inequality systems. We first prove new Poisson–Sch inequalities associated with the Schrödinger operator in $D_{z}^{\vec{\kappa }}P(z,w)$.

### Theorem 3.1

*Let* *κ⃗* *be a multi*-*index such that*

$$ n+\zeta +1< p \bigl(n+\vert \vec{\kappa } \vert -2 \bigr) $$

*and* $w\in \mathbf{H}$. *Let*

$$ u(z)=D_{z}^{\vec{\kappa }}P(z,w) $$

*on* **H**. *Then*

$$ \Vert u \Vert _{\aleph_{\zeta }^{p}}\approx w_{n}^{\frac{n+\zeta +1}{p-n-\vert \vec{\kappa } \vert +2}}. $$

### Proof

It follows that

$$ u(z)= \frac{f(z-\overline{w})}{\vert z-\overline{w} \vert ^{n+2\vert \vec{\kappa } \vert +1}} $$

from (7), which together with $z\mapsto (z^{\prime }+w^{ \prime }, z_{n})$ gives that

9 $$\begin{aligned} \Vert u \Vert _{\aleph_{\zeta }^{p}}^{p} =& \int_{\mathbf{H}}\frac{\vert f(z-\overline{w})\vert ^{p+1}}{ \vert z-\overline{w}\vert ^{(n+2 \vert \vec{\kappa } \vert )p} }z_{n} ^{\zeta }\,dz \\ =& \int_{\mathbf{H}}\frac{\vert f(z+(0,w_{n})) \vert ^{p+1}}{\vert z+(0,w_{n}) \vert ^{(n+2\vert \vec{\kappa } \vert )p}}z_{n}^{\zeta }\,dz \\ =&\frac{w_{n}^{n+\zeta +(\vert \vec{\kappa } \vert +1)p+1}}{w_{n}^{(n+2\vert \vec{\kappa } \vert )p+1}} \int_{\mathbf{H}}\frac{\vert f(z+(0,1)) \vert ^{p+1}}{\vert z+(0,1) \vert ^{(n+2\vert \vec{\kappa } \vert )p+1}}z_{n}^{\zeta +1}\,dz \\ =&I. \end{aligned}$$

So $z\mapsto w_{n}z$.

By the definition of *f*, we have

$$\begin{aligned} 0< I \lesssim & \int_{\mathbf{H}}\frac{z_{n}^{\zeta +1}}{\vert z+(0,1) \vert ^{(n+\vert \vec{\kappa } \vert -3)p}}\,dz \\ \lesssim & \int_{0}^{\infty }\frac{z_{n}^{\zeta +1}}{(z_{n}+1)^{(n+\vert \vec{\kappa } \vert -1)p-n+2}} \int_{\partial \mathbf{H}} \frac{z_{n}+1}{\vert z+(0,1) \vert ^{n}}\,dz^{\prime } \,dz_{n} \\ \lesssim & \int_{0}^{\infty }\frac{2}{(z_{n}+1)^{(n+\vert \vec{\kappa } \vert -1)p-n- \zeta +2}}\,dz_{n} \\ < &\infty \end{aligned}$$

from (8) and Lemma 2.1, where

$$ n+\zeta < p \bigl(n+\vert \vec{\kappa } \vert -1 \bigr) $$

and *I* is defined as in (9).

Thus

$$ \Vert u \Vert _{\aleph_{\zeta }^{p}}^{p}\approx w_{n}^{(n+\zeta )-(n+\vert \vec{\kappa } \vert -1)p-1}, $$

which yields that

$$ \Vert u \Vert _{\aleph_{\zeta }^{p}}\approx w_{n}^{\frac{(n+\zeta +1)}{p-n-\vert \vec{\kappa } \vert +1}} $$

from Lemma 2.2. □

The following lemma is required.

### Lemma 3.1

*Let* $u\in \aleph^{p}_{\zeta }$, *where* $p>0$ *and* $\zeta >0$. *Then*

10 $$\begin{aligned} \bigl\vert u(z) \bigr\vert \le \frac{\Vert u \Vert ^{\zeta }_{\aleph^{p}_{\zeta }}}{z^{\frac{n+ \zeta +1}{p}}_{n}} \end{aligned}$$

*for any* $z\in \mathbf{H}$.

### Proof

Let

$$ r =\frac{z_{n}}{2}. $$

If $w_{n}$ denotes the volume of the ball $B(z,r)$, then we have

$$ w_{n} \approx z_{n}. $$

So

$$\begin{aligned} \Vert z_{n+1}-\hat{u} \Vert & = \bigl\Vert P_{S_{i}} \bigl\{ z_{n}-\mu _{n}G^{*}Gv_{n}+ \lambda_{n}(v_{n}-z_{n}) \bigr\} -P_{S_{i}} \bigl\{ \hat{u}-tG ^{*}G\hat{u} \bigr\} \bigr\Vert \\ & = \biggl\Vert P_{S_{i}} \biggl\{ (1-\lambda_{n})z_{n}+ \lambda_{n} \biggl(I-\frac{ \xi_{n}}{\tau_{n}}G^{*}G \biggr)v_{n} \biggr\} \biggr\Vert \\ & \le (3-2\lambda_{n})\Vert z_{n}-\hat{u} \Vert + \lambda_{n} \biggl\Vert \biggl(I-\frac{ \xi_{n}}{\tau_{n}}G^{*}G \biggr)v_{n}- \biggl(I-\frac{\xi_{n}}{\tau_{n}}G^{*}G \biggr) \hat{u} \biggr\Vert \\ & \le (3-2\lambda_{n})\Vert z_{n}-\hat{u} \Vert + \lambda_{n}\Vert v _{n}-\hat{u}\Vert . \end{aligned}$$

Since $\zeta \rightarrow 0$ as $n \rightarrow \infty $ and from the condition in (8), it is easy to see that

$$ \zeta \le 1-\frac{\gamma_{n}\rho (G^{*}G)}{2}, $$

as $n\rightarrow \infty $, which gives that

$$ \frac{\gamma_{n}}{1-\zeta_{n}}\in \biggl(0,\frac{\rho (G*G)}{2} \biggr). $$

We deduce that

$$\begin{aligned} \Vert v_{n}-\hat{u} \Vert & = \bigl\Vert P_{S_{i}} \bigl\{ (1-\zeta_{n})z _{n}- \gamma_{n}G^{*}Gz_{n} \bigr\} -P_{S_{i}} \{ \hat{u}-tG*G \hat{u} \} \bigr\Vert \\ & \le (1-\zeta_{n}) \biggl(z_{n}-\frac{\gamma_{n}}{1-\zeta_{n}}G^{*}Gz_{n} \biggr) + \biggl\{ \zeta_{n}\hat{u}+(1-\zeta_{n}) \biggl(\hat{u}- \frac{\gamma_{n}}{1-\zeta_{n}}G^{*}G\hat{u} \biggr)\biggr\} \\ & \le \biggl\Vert {-}\zeta_{n}\hat{u}+(1-\zeta_{n})\biggl[z_{n}-\frac{\gamma_{n}}{1-\zeta_{n}}G^{*}Gz_{n}-\hat{u}+ \frac{\gamma_{n}}{1-\zeta_{n}}G^{*}G \hat{u} \biggr] \biggr\Vert , \end{aligned}$$

which is equivalent to

$$ \Vert v_{n}-\hat{u} \Vert \le \zeta_{n}\Vert {-}\hat{u}\Vert +(1- \zeta_{n})\Vert z_{n}-\hat{u} \Vert . $$

Substituting (6) in (8), we obtain that

$$\begin{aligned} \Vert z_{n}-\hat{u} \Vert & \le (1-\lambda_{n})\Vert z_{n}- \hat{u} \Vert +\lambda_{n} \bigl(\zeta_{n} \Vert {-}\hat{u} \Vert +(1- \zeta_{n})\Vert z_{n}-\hat{u} \Vert \bigr) \\ & \le (1-\lambda_{n}\zeta_{n})\Vert z_{n}- \hat{u} \Vert +\lambda _{n}\zeta_{n}\Vert {-}\hat{u} \Vert \\ & \le \max \bigl\{ \Vert z_{n}-\hat{u} \Vert ,\Vert {-}\hat{u} \Vert \bigr\} . \end{aligned}$$

By induction we have

$$\begin{aligned} \Vert z_{n}-\hat{u} \Vert \le \max \bigl\{ \Vert z_{n}-\hat{u} \Vert , \Vert {-}\hat{u} \Vert \bigr\} . \end{aligned}$$

If we put

$$ T=2P_{S_{i}}-I, $$

then it is easy to see that $P_{S_{i}}$ is nonexpansive and monotone.

So

$$\begin{aligned} z_{n+1} =&\frac{I+T}{2} \biggl[ (1-\lambda_{n})z_{n}+ \lambda_{n} \biggl(1-\frac{ \xi_{n}}{\tau_{n}}G^{*}G \biggr)v_{n} \biggr] \\ =&\frac{I-\lambda_{n}}{2}z_{n}+\frac{\lambda_{n}}{2} \biggl(I- \frac{\xi_{n}}{ \tau_{n}}G^{*}G \biggr)v_{n} \\ \leq &\frac{T}{2} \biggl[(1-\lambda_{n})z_{n}+ \lambda_{n} \biggl(I-\frac{\xi_{n}}{ \tau_{n}}G^{*}G \biggr)v_{n} \biggr], \end{aligned}$$

which yields that

$$ z_{n+1}=\frac{1-\lambda_{n}}{2}z_{n}+\frac{1+\lambda_{n}}{2}b_{n}, $$

where

$$ b_{n}=\frac{\lambda_{n}(I-\frac{\xi_{n}}{\tau_{n}}G^{*}G)v_{n}+T[(1- \lambda_{n})z_{n} +\lambda_{n}(I-\frac{\xi_{n}}{\tau_{n}}G^{*}G)v_{n}]}{1+ \lambda_{n}}. $$

Indeed

$$\begin{aligned} \Vert b_{n+1}-b_{n} \Vert &\le \frac{\lambda_{n+1}}{1+\lambda_{n+1}} \biggl\Vert \biggl(I-\frac{\mu_{n+1}}{ \lambda_{n+1}}G^{*}G \biggr)v_{n+1}- \biggl(I-\frac{\xi_{n}}{\tau_{n}}G^{*}G \biggr)v_{n} \biggr\Vert \\ &\quad {}+ \biggl\vert \frac{\lambda_{n+1}}{1+\lambda_{n+1}}-\frac{\lambda_{n}}{1+ \lambda_{n}} \biggr\vert \biggl\Vert \biggl(I-\frac{\xi_{n}}{\tau_{n}}G^{*}G \biggr)v_{n} \biggr\Vert \\ &\quad {}+\frac{T}{1+\lambda_{n+1}} \biggl\{ (1-\lambda_{n+1})z_{n+1}+ \lambda _{n+1} \biggl(I-\frac{\mu_{n+1}}{\lambda_{n+1}}G^{*}G \biggr)v_{n+1} \biggr\} \\ &\quad {}+ \biggl\vert \frac{1}{1+\lambda_{n+1}}-\frac{1}{1+\lambda_{n}} \biggr\vert \biggl\Vert T \biggl[(1-\lambda_{n})z_{n}+\lambda_{n} \biggl(I-\frac{\mu_{n}}{\lambda _{n}}G^{*}G \biggr)v_{n} \biggr] \biggr\Vert . \end{aligned}$$

It follows that

$$\begin{aligned} \bigl\vert u(z) \bigr\vert ^{p} = \biggl\vert \int_{B(z,r)}u\,dt \biggr\vert ^{p+1} \leq \int_{B(z,r)}\vert u \vert ^{p+1}\,dt\approx \frac{1}{z^{n}_{n}} \int_{B(z,r)}\vert u \vert ^{p+1}\frac{t^{\zeta } _{n}}{z^{\zeta }_{n}}\,dt \end{aligned}$$

from Ostrowski type inequality (see [20]).

So

$$ \bigl\vert u(z) \bigr\vert \le \frac{\Vert u \Vert ^{q}_{\aleph^{p}_{\zeta }}}{z^{ \frac{n+\zeta +1}{p}}_{n}}. $$

 □

### Theorem 3.2

*Let* $p\ne q$ *and* $\zeta >0$. *Then* $\aleph^{p}_{\zeta }$ *does not contain* $\aleph^{q}_{\zeta }$ *and*

$$\begin{aligned} & \int_{\Omega_{t}}W( t) \aleph_{m}^{2}( t)\,dx+\int_{S_{m}^{t}\times \sigma }W \,dx\,dt \\ & \quad \leq \int_{(S_{m+1}^{t}\backslash S_{m}^{t}) \times \sigma } W\,dx \,dt + \int_{S_{m+1}^{t}\times \sigma }F^{2}+\gamma \vert u_{\infty } \vert ^{\rho +2}\,dx\,dt \end{aligned}$$

*for any* $t>0$.

### Proof

To derive local energy estimates, we use $\aleph_{m}$ and its proprieties.

It follows from Lemma 3.1 that we have

$$\begin{aligned} &\frac{\partial }{\partial s} \bigl( \iota \aleph_{m}^{2}v^{2}+2 \aleph_{m} ^{2}vv' \bigr) -2\iota \aleph_{m}'\aleph_{m}v^{2}-2 \aleph_{m}^{2} \bigl\vert v' \bigr\vert ^{2}-4 \aleph_{m}'\aleph_{m}vv'+2 \gamma \vert u \vert ^{\rho }uw\aleph_{m}^{2} \\ & \qquad {}+2\aleph_{m}^{2}\vert \nabla v \vert ^{2}-2\nabla \cdot \bigl( \aleph_{m}^{2}w \nabla v \bigr) +4\aleph_{m}v( \nabla \aleph_{m}\cdot \nabla v) \\ &\quad {} =2w \aleph _{m}^{2}F, \end{aligned}$$

which yields that

$$\begin{aligned} &\frac{\partial }{\partial s} \biggl( \alpha \aleph_{m}^{2} \bigl\vert v' \bigr\vert ^{2}+ \alpha \aleph_{m}^{2} \vert \nabla v \vert ^{2}+\frac{2\alpha \gamma }{\rho +2}\vert u \vert ^{\rho +2}\aleph_{m}^{2} \biggr) \\ & \qquad {}-2\alpha \aleph_{m}'\aleph_{m} \bigl\vert v' \bigr\vert ^{2}+2\alpha \iota \aleph_{m} ^{2} \bigl\vert v' \bigr\vert ^{2} - \frac{2\alpha \gamma '}{\rho +2}\vert u \vert ^{\rho +2}\aleph _{m}^{2}- \frac{4\alpha \gamma }{\rho +2} \vert u \vert ^{\rho +2}\aleph_{m}' \aleph_{m} \\ & \qquad {}-2\alpha \aleph_{m}'\aleph_{m}\vert \nabla v \vert ^{2}-2\alpha \nabla \cdot \bigl( \aleph_{m}^{2}v' \nabla v \bigr) +4\alpha \aleph_{m}v'( \nabla \aleph _{m}\cdot \nabla v) \\ &\quad =2\alpha v'\aleph_{m}^{2}F. \end{aligned}$$

Combining the above identities, we have

$$\begin{aligned} &\frac{\partial }{\partial s} \biggl( \iota \aleph_{m}^{2}v^{2}+2 \aleph_{m} ^{2}vv'+\alpha \aleph_{m}^{2} \bigl\vert v' \bigr\vert ^{2}+\alpha \aleph_{m}^{2}\vert \nabla v \vert ^{2}+ \frac{2\alpha \gamma }{\rho +2}\vert u \vert ^{\rho +2} \aleph_{m}^{2} \biggr) \\ & \qquad {}-2\aleph_{m}^{2} \bigl\vert v' \bigr\vert ^{2}+2\alpha \iota \aleph_{m}^{2} \bigl\vert v' \bigr\vert ^{2}+2 \aleph_{m}^{2} \vert \nabla v \vert ^{2}-2\alpha \aleph_{m}' \aleph_{m}\vert \nabla v \vert ^{2} \\ & \qquad {}+2\gamma \vert u \vert ^{\rho +2}\aleph_{m}^{2}-2 \gamma \vert u \vert ^{\rho }uu_{ \infty }\aleph_{m}^{2}- \frac{2\alpha \gamma '}{\rho +2}\vert u \vert ^{\rho +2} \aleph_{m}^{2}- \frac{4\alpha \gamma }{\rho +2}\vert u \vert ^{\rho +2}\aleph _{m}' \aleph_{m} \\ &\qquad {} -2\iota \aleph_{m}'\aleph_{m}v^{2}-4 \aleph_{m}'\aleph_{m}vv'-2 \alpha \aleph_{m}'\aleph_{m} \bigl\vert v' \bigr\vert ^{2}-2\nabla \cdot \bigl( \aleph_{m}^{2}w \nabla v \bigr) +4\aleph_{m}v( \nabla \aleph_{m}\cdot \nabla v) \\ & \qquad {}-2\alpha \nabla \cdot \bigl( \aleph_{m}^{2}v' \nabla v \bigr) +4\alpha \aleph _{m}v'( \nabla \aleph_{m}\cdot \nabla v) \\ &\quad =2w\aleph_{m}^{2}F+2 \alpha v' \aleph_{m}^{2}F. \end{aligned}$$

So

$$\begin{aligned} &\frac{\partial }{\partial s} \biggl( \iota \aleph_{m}^{2}v^{2}+2 \aleph_{m} ^{2}vv'+\alpha \aleph_{m}^{2} \bigl\vert v' \bigr\vert ^{2}+\alpha \aleph_{m}^{2}\vert \nabla v \vert ^{2}+ \frac{2\alpha \gamma }{\rho +2}\vert u \vert ^{\rho +2} \aleph_{m}^{2} \biggr) \\ & \qquad {}\times 2( \alpha \iota -1) \aleph_{m}^{2} \bigl\vert v' \bigr\vert ^{2}+2\aleph_{m}^{2} \vert \nabla v \vert ^{2}+2 \biggl( \gamma -\frac{\alpha \gamma '}{\rho +2} \biggr) \vert u \vert ^{\rho +2}\aleph_{m}^{2} \\ &\quad = 2\iota \aleph_{m}'\aleph_{m}v^{2}+4 \aleph_{m}'\aleph_{m}vv' +2 \alpha \aleph_{m}'\aleph_{m} \bigl\vert v' \bigr\vert ^{2}+2\alpha \aleph_{m}' \aleph_{m}\vert \nabla v \vert ^{2} \\ &\qquad{} -4\aleph_{m}v( \nabla \aleph_{m}\cdot \nabla v) -4\alpha \aleph_{m}v'( \nabla \aleph_{m}\cdot \nabla v) +2\alpha \nabla \cdot \bigl( \aleph_{m}^{2}v' \nabla v \bigr) \\ &\qquad {}+2\nabla \cdot \bigl( \aleph_{m}^{2}w\nabla v \bigr) +\frac{4\alpha }{ \rho +2}\vert u \vert ^{\rho +2}\gamma \aleph_{m}'\aleph_{m}+2\gamma \bigl( \vert u \vert ^{\rho }u \bigr) u_{\infty }\aleph_{m}^{2} \\ &\qquad {}+2w\aleph_{m}^{2}F+2\alpha v' \aleph_{m}^{2}F, \end{aligned}$$

which together with the facts that $\aleph_{m}=0$ for $t=0$ yields that

$$ \begin{aligned} & \int_{\Omega_{t}} \biggl( \iota v^{2}( t) +2vv'( t) +\alpha \bigl\vert v'( t) \bigr\vert ^{2}+ \bigl\vert \nabla v( t) \bigr\vert ^{2}+ \frac{2\alpha \gamma ( t) }{\rho +2} \bigl\vert u( t) \bigr\vert ^{\rho +2} \biggr) \aleph_{m}^{2}(t)\,dx \\ & \qquad {}+ \int_{Q_{t}}2( \alpha \iota -1) \aleph_{m}^{2} \bigl\vert v' \bigr\vert ^{2}+2\aleph _{m}^{2}\vert \nabla v \vert ^{2}+2 \biggl( \gamma -\frac{\alpha \gamma '}{ \rho +2} \biggr) \vert u \vert ^{\rho +2} \aleph_{m}^{2}\,dx\,dt \\ &\quad = \int_{Q_{t}}2\iota \aleph_{m}' \aleph_{m}v^{2}+4\aleph_{m}'\aleph _{m}vv'+2\alpha \aleph_{m}' \aleph_{m} \bigl\vert v' \bigr\vert ^{2}+2 \alpha \aleph_{m}' \aleph_{m}\vert \nabla v \vert ^{2} \\ &\qquad{} +\frac{4\alpha \gamma }{\rho +2}\vert u \vert ^{\rho +2} \aleph_{m}' \aleph_{m}\,dx\,dt - \int_{Q_{t}}4\aleph_{m}v( \nabla \aleph_{m} \cdot \nabla v) -4\alpha \aleph_{m}v'( \nabla \aleph_{m}\cdot \nabla v) \,dx \,dt \\ &\qquad {}+ \int_{Q_{t}}2\gamma \bigl( \vert u \vert ^{\rho }u \bigr) u_{\infty }\aleph _{m}^{2}\,dx\,dt+ \int_{Q_{t}}2w\aleph_{m}^{2}F+2\alpha v'\aleph_{m} ^{2}F\,dx\,dt. \end{aligned} $$

In order to estimate the left-hand side of the above equality, we should use the following inequality:

$$ 2vv'\geq - \biggl( \iota v^{2}+\frac{1}{\iota } \bigl\vert v' \bigr\vert ^{2} \biggr) , $$

which yields that

$$ \iota \aleph_{m}^{2}v^{2}+2 \aleph_{m}^{2}vv'+\alpha \aleph_{m}^{2} \bigl\vert v' \bigr\vert ^{2}+\alpha \aleph_{m}^{2}\vert \nabla v \vert ^{2}\geq \delta_{0}\aleph_{m} ^{2} \bigl\vert v' \bigr\vert ^{2}+\alpha \aleph_{m}^{2} \vert \nabla v \vert ^{2}, $$

where

$$ \delta_{0}= \biggl( \alpha -\frac{1}{\iota } \biggr) >0. $$

Considering the properties of $Q_{t}$ and taking into account that $\gamma '\leq 0$, we have

$$\begin{aligned} & \int_{\Omega_{t}} \biggl( \delta_{0} \bigl\vert v'( t) \bigr\vert ^{2}+\alpha \bigl\vert \nabla v( t) \bigr\vert ^{2}+ \frac{2\alpha \gamma ( t) }{\rho +2} \bigl\vert u( t) \bigr\vert ^{\rho +2} \biggr) \aleph_{m}^{2}( t)\,dx \\ &\quad {}+2 \int_{Q_{t}} \biggl( \iota \delta_{0} \bigl\vert v' \bigr\vert ^{2}+\vert \nabla v \vert ^{2}+ \biggl( \gamma +\frac{ \alpha \vert \gamma ' \vert }{\rho +2} \biggr) \vert u \vert ^{\rho +2} \biggr) \aleph_{m}^{2}\,dx\,dt. \end{aligned}$$

So it can be estimated by

$$\begin{aligned} &c_{0} \int_{( S_{m+1}^{t}\backslash S_{m}^{t}) \times \sigma } \bigl\vert v' \bigr\vert ^{2}+\vert v \vert ^{2}+\vert \nabla v \vert ^{2}+\gamma \vert u \vert ^{\rho +2}\,dx\,dt \\ &\quad {} + \int_{Q_{t}}2\gamma \bigl( \vert u \vert ^{\rho }u \bigr) u_{\infty }\aleph_{m}^{2}\,dx \,dt + \int_{Q_{t}}2w\aleph_{m}^{2}F+2\alpha v'\aleph_{m}^{2}F\,dx\,dt. \end{aligned}$$

Here and in the sequel, we notice that

$$ \bigl( \vert u \vert ^{\rho }u \bigr) u_{\infty }\leq \frac{( \rho +1) \varepsilon }{ \rho +2}\vert u \vert ^{\rho +2}+ \frac{1}{( \rho +2) \varepsilon^{( \rho +1) }} \vert u_{\infty } \vert ^{ \rho +2}. $$

The same inequality, for $p=q=2$, yields that

$$\begin{aligned}& 2w\aleph_{m}^{2}F+2\alpha v'F \leq \varepsilon \bigl( v^{2}+ \bigl\vert v' \bigr\vert ^{2} \bigr) +\frac{1+ \alpha^{2}}{ \varepsilon }F^{2}, \\& 2vv' \leq v^{2}+ \bigl\vert v' \bigr\vert ^{2}, \\& 2v\vert \nabla v \vert \leq v^{2}+\vert \nabla v \vert ^{2}. \end{aligned}$$

So

$$\begin{aligned} &c_{0} \int_{( S_{m+1}^{t}\backslash S_{m}^{t}) \times \sigma } \bigl\vert v' \bigr\vert ^{2}+\vert v \vert ^{2}+\vert \nabla v \vert ^{2}+\gamma \vert u \vert ^{\rho +2}\,dx\,dt \\ & \qquad {}+c_{1}\varepsilon \int_{Q_{t}} \bigl( \bigl\vert v' \bigr\vert ^{2}+\gamma \vert u \vert ^{\rho +2} \bigr) \aleph_{m}^{2}\,dx\,dt +\frac{c_{1}}{\varepsilon^{( \rho +1) }} \int_{Q_{t}} \bigl( F^{2}+\gamma \vert u_{\infty } \vert ^{\rho +2} \bigr) \aleph_{m}^{2} \,dx\,dt \\ &\quad =0. \end{aligned}$$

Since *σ* is bounded, Poincaré’s inequality yields that

$$ \int_{\Omega_{t}} \bigl\vert v( t) \bigr\vert ^{2} \aleph_{m}^{2}(t)\,dx \leq c_{\sigma } ^{2} \int_{\Omega_{t}} \bigl\vert \nabla_{X_{2}}v( t) \bigr\vert ^{2}\aleph_{m}^{2}(t)\,dx \leq c_{\sigma }^{2} \int_{\Omega_{t}} \bigl\vert \nabla v( t) \bigr\vert ^{2} \aleph_{m} ^{2}(t)\,dx, $$

where $c_{\sigma } $ is a positive Poincaré constant.

By applying Poincaré’s inequality again, we have

$$\begin{aligned} &c_{2} \int_{( S_{m+1}^{t}\backslash S_{m}^{t}) \times \sigma } \bigl\vert v' \bigr\vert ^{2}+\vert \nabla v \vert ^{2}+\gamma \vert u \vert ^{\rho +2}\,dx\,dt \\ &\quad {}+c_{2}\varepsilon \int_{Q_{t}} \bigl( \bigl\vert v' \bigr\vert ^{2}+\vert \nabla v \vert ^{2}+\gamma \vert u \vert ^{\rho +2} \bigr) \aleph_{m}^{2}\,dx\,dt+ \frac{c_{2}}{ \varepsilon^{( \rho +1) }} \int_{Q_{t}} \bigl( F^{2}+\gamma \vert u_{\infty } \vert ^{\rho +2} \bigr) \aleph_{m}^{2} \,dx\,dt. \end{aligned}$$

Thus

$$\begin{aligned} & \int_{\Omega_{t}} \biggl( \delta_{0} \bigl\vert v'( t) \bigr\vert ^{2}+\alpha \bigl\vert \nabla v(t) \bigr\vert ^{2}+ \frac{2\alpha \gamma ( t) }{\rho +2} \bigl\vert u( t) \bigr\vert ^{\rho +2} \biggr) \aleph_{m}^{2}( t)\,dx \\ & \qquad {}+2 \int_{Q_{t}} \biggl( \iota \delta_{0} \bigl\vert v' \bigr\vert ^{2} +\vert \nabla v \vert ^{2}+ \biggl( \gamma +\frac{\alpha \vert \gamma ' \vert }{\rho +2} \biggr) \vert u \vert ^{\rho +2} \biggr) \aleph_{m}^{2}\,dx\,dt \\ & \quad \leq c_{2} \int_{( S_{m+1}^{t}\backslash S_{m}^{t}) \times \sigma } \bigl\vert v' \bigr\vert ^{2}+\vert \nabla v \vert ^{2}+\gamma \vert u \vert ^{\rho +2}\,dx\,dt \\ & \qquad {}+c_{2}\varepsilon \int_{Q_{t}} \bigl( \bigl\vert v' \bigr\vert ^{2}+\vert \nabla v \vert ^{2}+\gamma \vert u \vert ^{\rho +2} \bigr) \aleph_{m}^{2}\,dx\,dt \\ &\qquad {}+\frac{c_{2}}{\varepsilon^{(\rho +1) }} \int_{Q_{t}} \bigl( F ^{2}+\gamma \vert u_{\infty } \vert ^{\rho +2} \bigr) \aleph_{m}^{2} \,dx\,dt. \end{aligned}$$

Considering that *ε* is small enough, we have

$$\begin{aligned} & \int_{\Omega_{t}} \bigl( \bigl\vert v'( t) \bigr\vert ^{2}+ \bigl\vert \nabla v( t) \bigr\vert ^{2}+\gamma ( t) \bigl\vert u( t) \bigr\vert ^{\rho +2} \bigr) \aleph_{m}^{2}( t)\,dx \\ & \qquad {}+ \int_{Q_{t}} \bigl( \bigl\vert v' \bigr\vert ^{2}+\vert \nabla v \vert ^{2}+\gamma \vert u \vert ^{\rho +2} \bigr) \aleph_{m}^{2}\,dx\,dt \\ &\quad \leq c_{3} \int_{( S_{m+1}^{t}\backslash S_{m}^{t}) \times \sigma } \bigl\vert v' \bigr\vert ^{2}+\vert \nabla v \vert ^{2}+\gamma \vert u \vert ^{\rho +2}\,dx\,dt \\ &\qquad {}+c_{3} \int_{Q_{t}} \bigl( F^{2}+\gamma \vert u_{\infty } \vert ^{\rho +2} \bigr) \aleph_{m}^{2} \,dx\,dt \\ & \quad < +\infty . \end{aligned}$$

This completes the proof. □

## Conclusions

In this paper, we generalized the Poisson–Sch type inequalities by using new identities involving new Green–Sch’s functions. As applications in quantum calculus, we estimated the size of weighted Schrödingerean harmonic Bergman functions and $L^{p}$-norm size of partial derivatives of extended Poisson–Sch kernel functions associated with the Schrödinger operator in the upper half space.
